# Soluble beta amyloid evokes alteration in brain norepinephrine levels: role of nitric oxide and interleukin-1

**DOI:** 10.3389/fnins.2015.00428

**Published:** 2015-11-05

**Authors:** Maria G. Morgese, Marilena Colaianna, Emanuela Mhillaj, Margherita Zotti, Stefania Schiavone, Palma D'Antonio, Andrew Harkin, Valentina Gigliucci, Patrizia Campolongo, Viviana Trezza, Angelo De Stradis, Paolo Tucci, Vincenzo Cuomo, Luigia Trabace

**Affiliations:** ^1^Department of Clinical and Experimental Medicine, University of FoggiaFoggia, Italy; ^2^Department of Pathology and Immunology, University of GenevaGeneva, Switzerland; ^3^Department of Physiology and Pharmacology, La Sapienza, University of RomeRome, Italy; ^4^Neuropsychopharmacology Research Group, School of Pharmacy and Pharmaceutical Sciences and Trinity College Institute of Neuroscience, Trinity College DublinDublin, Ireland; ^5^Section of Biomedical Sciences and Technologies, Department of Science, University “Roma Tre,”Rome, Italy; ^6^Department of Bio Agro-Food Sciences, The Institute of Sustainable Plant Protection, National Research CouncilBari, Italy

**Keywords:** soluble beta amyloid, norepinephrine, nitric oxide, interleukin-1, prefrontal cortex, hippocampus, nucleus accumbens

## Abstract

Strong evidence showed neurotoxic properties of beta amyloid (Aβ) and its pivotal role in the Alzheimer's disease (AD) pathogenesis. Beside, experimental data suggest that Aβ may have physiological roles considering that such soluble peptide is produced and secreted during normal cellular activity. There is now suggestive evidence that neurodegenerative conditions, like AD, involve nitric oxide (NO) in their pathogenesis. Nitric oxide also possess potent neuromodulatory actions in brain regions, such as prefrontal cortex (PFC), hippocampus (HIPP), and nucleus accumbens (NAC). In the present study, we evaluated the effect of acute Aβ injection on norepinephrine (NE) content before and after pharmacological manipulations of nitrergic system in above mentioned areas. Moreover, effects of the peptide on NOS activity were evaluated. Our data showed that 2 h after i.c.v. soluble Aβ administration, NE concentrations were significantly increased in the considered areas along with increased iNOS activity. Pre-treatment with NOS inhibitors, 7-Nitroindazole (7-NI), and N6-(1-iminoethyl)-L-lysine-dihydrochloride (L-NIL), reversed Aβ-induced changes. Ultimately, pharmacological block of interleukin1 (IL-1) receptors prevented NE increase in all brain regions. Taken together our findings suggest that NO and IL-1 are critically involved in regional noradrenergic alterations induced by soluble Aβ injection.

## Introduction

Amyloid beta peptide (Aβ) is the main component of the amyloid plaques, one of the neuropathological hallmarks of Alzheimer's disease (AD; Di Carlo et al., 2012). It has been hypothesized that the peptide accumulation initiated by a pathogenic cascade ultimately leads to AD (Hardy and Selkoe, 2002). However, mounting evidence suggest that Aβ, besides its well accepted neurotoxic activity, may have physiological roles (Mura et al., 2010a). The peptide, in its soluble form, is produced and secreted during normal cellular activity (Kar et al., 2004) and is able to modulate synaptic activity in the absence of neurotoxicity (Grilli et al., 2010; Mura et al., 2010b). Physiologically, the neuromodulatory role of Aβ would be important for the right functioning of neurotransmitter systems; however, in pathological conditions, Aβ-modulating synaptic activity could trigger functional alterations on neurotransmission (Mura et al., 2012).

On the other hand, several hints point to the involvement of the free radical gas, nitric oxide (NO), in the pathogenesis of neurodegenerative conditions, such as AD (Guix et al., 2005; Fernandez et al., 2010). In the brain, NO is synthesized from L-Arginine (L-Arg) either by inducible nitric oxide synthase (iNOS) in microglia and astrocytes, or by constitutive NOS in neurons and endothelial cells (nNOS and eNOS, respectively). It has been suggested that NO produced by constitutive NOS is accountable for neuroprotection from Aβ-induced cell death, while NO release, following iNOS activation, plays a neurotoxic role (Puzzo et al., 2006). The three NOS isoforms have been proposed to mediate Aβ effects, producing elevated levels of NO, which could contribute to worsen the outcomes of the disease (Fernandez et al., 2010). Although NO behaves as a neurotoxic effector when produced in excessive amount, increasing evidence focus on its neuroprotective properties in mediating cellular transduction mechanisms, regulating neuronal plasticity (Palumbo et al., 2007) and suppressing neuronal apoptotic cell death (Contestabile and Ciani, 2004). By playing a potent neuromodulatory role in several brain regions, such as prefrontal cortex (PFC), hippocampus (HIPP), nucleus accumbens (NAC), and striatum (Trabace et al., 2004, 2007; Saulskaya et al., 2010; Bechade et al., 2011), NO has been presented either as neuroprotective or restorative in neurodegenerative conditions such as AD (Puzzo et al., 2005). Recent evidence linked Aβ effects to the nitrergic pathway; indeed, as suggested by experiments performed in mouse and human cells, Aβ caused a decrease in the activity of the soluble guanylate cyclase sinking NO signaling by binding to the cell surface receptor CD36 (Miller et al., 2010). Therefore, the enhancement of NO levels can be proposed as protective toward neurons from their degeneration and death.

Interestingly, there is several *in vivo* and *in vitro* evidence supporting the NO pathway's role in the mechanism of norepinephrine (NE)-induced neuroprotection (Lonart et al., 1992; Chen et al., 2006; Chen and Russo-Neustadt, 2007). Indeed, in primary hippocampal neuronal cell culture, NE stimulation leads to an increased activation of several pathways, whose function has been proposed to be dependent on NO availability. Furthermore, such activation seems to be decreased in presence of NOS inhibition (Chen and Russo-Neustadt, 2007). In addition, it has been shown that NE promotes cell survival both *in vivo* (Chen and Russo-Neustadt, 2005) and in the primary hippocampal neuronal cell culture (Chen and Russo-Neustadt, 2007). Previous reports also indicate that NE is an important player in innate immunosuppressive maintenance within the central nervous system (Feinstein et al., 2002; Heneka et al., 2002); it has been reported that NE holds anti-inflammatory properties by modulating microglial functions following the degeneration of aminergic locus coeruleus (LC) neurons induced in APP-transgenic mice by using the neurotoxin DSP4 (Heneka et al., 2010). In particular, it has been shown that NE, through the activation of β_2_ adrenoceptors present on microglial cells, is able to promote endocytosis and degradation of Aβ_42_ (Kong et al., 2010). Furthermore, NE can significantly reduce glial iNOS via activation of β_2_ adrenoceptors and cAMP increase (Kalinin et al., 2006).

Based on the above reported background, we were firstly interested in evaluating the interplay among Aβ peptide, noradrenergic, and nitrergic transmission. To this end, we evaluated effects of an acute soluble Aβ injection on noradrenergic neurotransmission; moreover, by using NOS inhibitors, we tested if such pharmacological manipulations of the nitrergic system were able to modulate the neurochemical effects induced by Aβ peptide in brain areas involved in AD, such as PFC, HIPP, and NAC.

In addition, we have previously demonstrated that intrahippocampal injection of soluble Aβ potently increases interleukin-1β levels (Sanz et al., 2009). Such cytokine has been reported to act at distance with NO mediating central NE effect (Hsieh et al., 2010). Thus, we further investigated the effect of the block of IL-1 receptors on NE content of above cited areas in soluble Aβ-treated animals.

## Materials and methods

### Animals

A total of 78 young adult male (250–300 g) Wistar rats (Harlan, S. Pietro al Natisone, Udine) were used in this study. They were housed at constant room temperature (22 ± 1°C) and relative humidity (55 ± 5%) under a 12 h light/dark cycle with *ad libitum* access to standard food and water. Procedures involving animals and their care were conducted in conformity with the institutional guidelines of the Italian Ministry of Health (D.L. 26/2014), the Guide for the Care and Use of Mammals in Neuroscience and Behavioral Research (National Research Council, 2004), the Directive 2010/63/EU of the European Parliament and of the Council of 22 September 2010 on the protection of animals used for scientific purposes. All procedures involving animals were conducted in accordance to ARRIVE guidelines. Animal welfare was daily monitored through the entire period of experimental procedures. No signs of distress were evidenced; anyway, all efforts were made to minimize the number of animals used and their suffering.

### Cannula implantation and Aβ administration

Cannula implantation was performed as previously described (Trabace et al., 2007). Briefly, unilateral 23-gauge stainless steel guide cannulae (Cooper's Needles, Birmingham, UK) were implanted using the following coordinates relative to bregma: AP = −0.5, L = +1.2, H = −3.0 with the incisor bar set at −3.3 mm, according to a stereotaxic atlas (Paxinos and Watson, 1998). On the day of the experiment, Aβ_(1-42)_ fresh solution, at a concentration previously tested (4 μM, in distilled water; Colaianna et al., 2010), was delivered via a 30-gauge needle lowered 2 mm below the cannula tip. The solution was then infused at a flow rate of 2 μL/min for 2 min 30 s through a Hamilton syringe connected to a microdialysis pump (CMA microdialysis, Sweden).

### Atomic force microscopy (AFM) and transmission electron microscopy (TEM)

Samples for AFM and TEM measurements were obtained by preparing a 4 μM solution of Aβ, both for the “initial state” and the “oligomers” phase. Freshly prepared 4 μM solution of Aβ was used immediately (“initial state solution”). To obtain Aβ oligomers, a different 4 μM solution of Aβ was prepared in 50 mM phosphate buffer and 150 mM NaCl (pH 7.4), and incubated for 24 h at 4°C (“oligomers” solution; Lambert et al., 1998).

For AFM analyses, some drops of the solution were deposited on a freshly cleaved mica substrate. After an incubation of 2 min, samples were washed with ultrapure water and dried for 30 min in a vacuum desiccator.

A Perception Atomic Force Microscope (Assing S.p.A., Italy) was used to record AFM images. Measurements were performed in air, working in the weak repulsive regime of contact mode. Gold coated Si_3_N_4_ cantilevers (model MSNL from Bruker Company, Massachusetts, USA) with a spring constant of 0.01 N/m and Silicon tips with a nominal apical radius of 2–12 nm were used for the topographic AFM images. Constant force images were acquired with a scan rate of about 8 s/row, at a resolution of 512 × 512 points.

For TEM analyses, a little drop (20 μL) of each incubated sample solution was applied to carbon coated copper/rhodium grid (400 mesh; TAAB Laboratories Equipment Ltd, Aldermaston, Berks, GB). The coated grid was floated for 2 min on the sample drop and rinsed with 200 mL of double distilled water. Negative staining was performed with 200 μL of 2% w/v uranyl acetate solution (TAAB Laboratories Equipment Ltd). After draining off the excess of staining solution by means of a filter paper, the specimen was transferred for examination in a Philips Morgagni 282D transmission electron microscope, operating at 60 kV. Electron micrographs of negatively stained samples were photographed on Kodak electron microscope film 4489 (Kodak Company, New York, USA).

### Pharmacological manipulation

Thirty minute before Aβ administration, NOS inhibitors 7-Nitroindazole (7-NI) (50 mg kg^−1^, Vinci Biochem, Florence, Italy), and N6-(1-iminoethyl)-L-lysine-dihydrochloride (L-NIL) (5 mg kg^−1^, Vinci Biochem, Florence, Italy) were dissolved in saline (1 ml kg^−1^, veh) and administered intraperitoneally (i.p.) (Trabace et al., 2007) according to the experimental assignment. Interleukin-1 receptor antagonist (IL-1ra, Sigma-Aldrich, Milan, Italy) was applied i.c.v. at a concentration of 100 ng 5 μl^−1^ per rat (Taepavarapruk and Song, 2010). Drugs were administered 30 min (NOS inhibitors) or 10 min (IL-1ra) before Aβ injection based upon our previous experience and following a survey of the relevant literature (Trabace et al., 2007; Choi et al., 2009).

### *Post-mortem* tissue analysis

Rats were euthanized and brains were immediately removed 2 h after Aβ administration. Thereafter, PFC, NAC, and HIPP were collected and stored frozen at −80°C until analyses. Samples were treated and NE and serotonin (5-HT) tissue concentrations were determined by using high performance liquid chromatography (HPLC) coupled with electrochemical detection as previously described (Trabace et al., 2011).

### HPLC analysis

NE and 5-HT concentrations were determined by HPLC coupled with an electrochemical detector (INTRO, Antec Leyden, The Netherlands). Separation was performed by a LC18 reverse phase column (hypersil, 150 × 3 mm, ODS 5 μm; Thermoscientific, Milan, Italy). The detection was accomplished by a Unijet cell (BASi, Kenilworth, U.K.) with a 6 mm diameter glassy carbon electrode at a working potential of 0.65 V vs. Ag/AgCl. The mobile phase used was 85 mM CH_3_COONa, 0.8 mM octane sulfonic acid, 0.3 mM EDTA, 15 mM NaCl, methanol 6%, in distilled water, buffered at pH 4.85. The flow rate was maintained by an isocratic pump (Shimadzu LC-10 AD, Kyoto, Japan) at 1 ml/min. Data were acquired and integrated using Chromeleon software (version 6.60, Dionex, San Donato Milanese, Italy).

### Quantification of NOS gene expression

#### RNA extraction

Total RNA was extracted from PFC, NAC, and HIPP by using Nucleospin RNA II kits (Macherey-Nagel, Dublin, Ireland) according to the manufacturer's instructions. Total RNA concentrations were measured with a NanoDrop® (Thermo Scientific, Dublin, Ireland) micro-volume spectrophotometer and all RNA samples were equalized with RNase-free water to the lowest detected concentration.

#### cDNA synthesis

cDNA was synthesized using the ABI High Capacity cDNA kit (Applied Biosystems, life technologies, Paisley, U.K.) as provided by the protocol. A 2X master-mix solution containing reverse transcription buffer, dNTPs, random primers, and MultiScribeTM reverse transcriptase was made up in RNase-free water and stored on ice. The reverse transcription reaction was performed in a thermocycler (PTC-200, MJ Research) with a three-step program: 10 min at 25°C followed by 120 min at 37°C and a final 5 min step at 85°C. cDNA samples were used immediately for real time PCR or stored at −20°C until needed.

#### Real-time PCR

Real-Time PCR was performed as multiplex using TaqMan® Gene Expression Assays in accordance with the manufacturer's instructions (Applied Biosystems, life technologies, Paisley, U.K.). The target genes nNOS (Rn00583793), iNOS (Rn00561646), and eNOS (Rn02132634) were identified by FAM-labeled probes, while the housekeeping gene of reference, β–actin (4352340E), was identified by a VIC-labeled probe. The mixture was prepared by adding a 1:5 dilution of the cDNA sample obtained from the previous reaction into a 96-well optical reaction plate (Applied Biosystems, life technologies, Paisley, U.K.), followed by 2X TaqMan® Universal PCR Master Mix (No AmpErase® UNG, Applied Biosystems, life technologies, Paisley, U.K.) and 20X primers for both the target gene and β-actin (to reach a final 1X concentration). Amplification reaction was achieved with a three-stage protocol: 2 min at 50°C followed by 10 min at 95°C, followed again by 40 repetitions of a two-step cycle composed by 15 s at 95°C (denaturation) and 1 min at 60°C (annealing and extension) (ABI Prism 7300 instrument, Applied Biosystems, life technologies, Paisley, U.K.). Data analysis was performed with the 7300 System Software (Applied Biosystems, life technologies, Paisley, U.K.) and RQ values (2-ΔΔCT, where CT is the threshold cycle) of the target genes relative to their own endogenous control were obtained. The RQ values were then converted into fold change values relative to control group. β-actin was used as the reference housekeeping gene in this study.

### Nitrite/nitrate (NOx) analysis

NOx levels were estimated using an ion chromatographic with conductometric detection method. The brain areas were suspended (1:20, w/v) in ultrapure water and placed at 70°C for 5 min. After cooling, the mixture was centrifuged for 5 min at 1500 × *g* at room temperature. Chromatographic determinations were performed on a Dionex DX-500 system (Dionex Corporation, Sunnyvale, CA, USA) injecting 25 μL of the supernatant. Chromatographic separations were accomplished using an IonPac AS9-HC column (250 × 4 mm) associated with a pre-column AG9-HC (4 mm, Dionex Corporation) eluted in isocratic mode at a flow rate of 1.0 mL/min. The mobile phase consisted of 9 mM sodium carbonate (total run time, 20 min) (Iammarino et al., 2012).

### Statistical analysis

Results were expressed as percentage of control (SHAM-veh) and mean ± S.E.M. Statistical analyses were performed using Graph Pad 5.0 (GraphPad Software, San Diego, CA) and Systat13 (Systat Software, Inc. San Jose, CA) for Windows. Neurotransmitter concentrations were analyzed using Two-way analysis of variance (ANOVA) followed by Bonferroni multiple comparisons test. NOx concentrations and NOS mRNA content were analyzed by using Student's *t*-test for independent comparison and Two-way ANOVA followed by Bonferroni multiple comparisons test, as required. Differences were considered statistically significant when *P*-value was less than 0.05.

## Results

### Evaluation of Aβ state through AFM and TEM measurements

**Figure 1:**
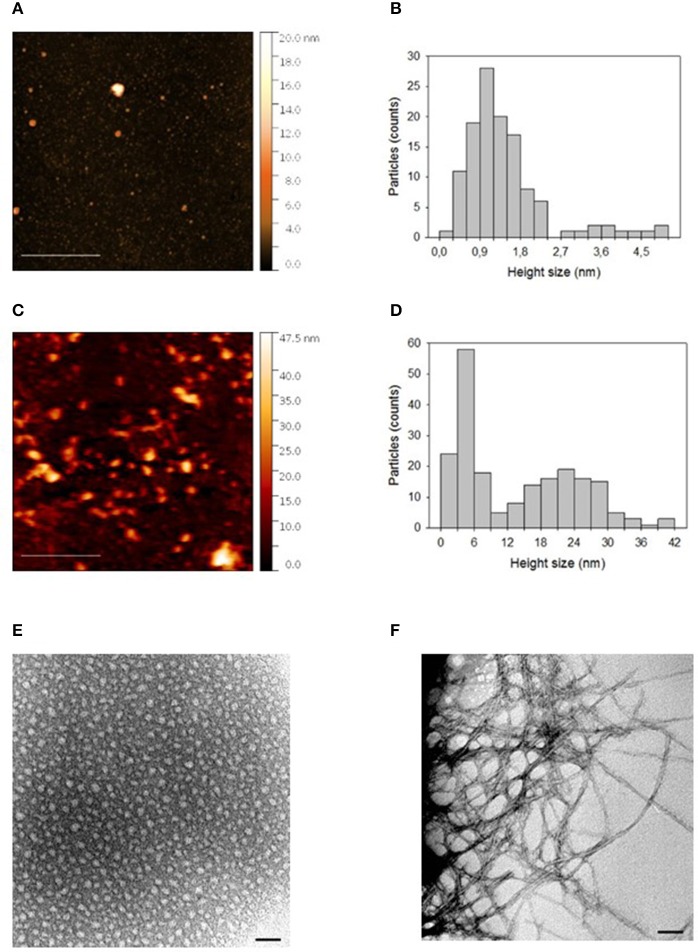
**(A)** AFM picture measured in contact mode by using Silicon tips having a nominal apical radius of 2–12 nm and **(B)** histogram representing the distribution of height size values of Aβ “initial state” solution deposited on a freshly cleaved mica disk. In **(C,D)** are reported the corresponding AFM picture and distribution of height size values for an “oligomer” solution of Aβ deposited on a freshly cleaved mica disk. The color scale reported on the right hand side of each AFM picture represents the height values of the measured structures. In **(E,F)**, TEM pictures of “initial state” and “oligomer state” solutions, respectively, are reported. The horizontal scale bar was 500 nm for AFM and 50 nm for TEM picture.

Figure 1 shows AFM images of “initial state” and “oligomers” solution of Aβ deposited on a freshly cleaved mica disk. It is evident that in both cases Aβ particles with different sizes are distributed on the mica substrate. The size of such particles was estimated by sphericity assumption, considering height values, because the lateral size is influenced by tip-particle convolution. In fact, Figure 1A showed that the “initial state” solution contained many small particles and very few larger particles. As indicated in Figure 1B, the distribution of height values of the small particles (height less than about 2.5 nm), peaked in the 0.9–1.2 nm range with a mean value of 1.2 ± 0.5 nm. The height of the few larger particles (height more than about 2.5 nm) was more uniformly distributed and was characterized by a mean value of 3.9 ± 0.6 nm. After 24 h incubation at 4°C and pH 7.4, particles assembled in larger aggregates, as shown in the AFM image of “oligomers” solution (Figure 1C). In this case, the aggregate sizes were characterized by a bimodal distribution (Figure 1D), where the two modal values were related to aggregates with a height size value smaller or larger than 9 nm, respectively. The smaller aggregates presented a mean height size of 4.4 ± 1.7 nm, whereas the mean height size of larger aggregates was 22.7 ± 6.7 nm. TEM analyses confirmed results obtained from AFM; indeed, as shown in Figure 1E, in the “initial state solution” the higher percentage of molecules were monomers, while in the “oligomer state solution” oligomers and fibrils were the most representative species (Figure 1F).

### Effects of nitrergic manipulation on regional NE and 5-HT concentrations in Aβ-injected rats

**Figure 2:**
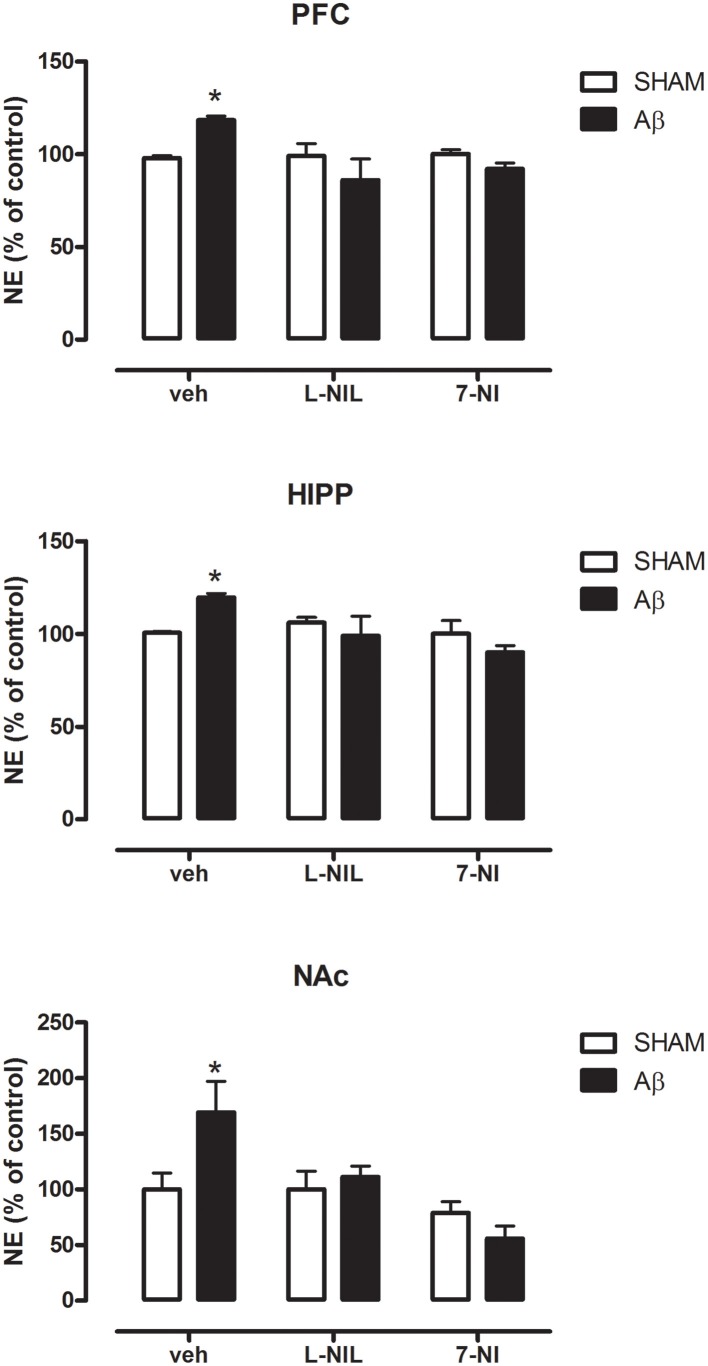
**NE levels in PFC, HIPP, and NAC of male Wistar rats 2 h after injection of water (5 μL i.c.v.; SHAM) or Aβ (4 μM, 5 μL i.c.v.; Aβ)**. Animals were pre-treated with vehicle (saline, veh, 1 ml kg^−1^), L-NIL (5 mg kg^−1^, i.p.), or 7-NI (50 mg kg^−1^, i.p.), 30 min before i.c.v. administration. Data are expressed as percentage of control (*n* = 6–7 per group; Two-way ANOVA with Bonferroni correction, ^*^P < 0.05).

As shown in Figure 2, NE concentrations were higher in Aβ-treated when compared to SHAM rats in all of the three areas investigated (Two-way ANOVA with Bonferroni correction, PFC:*P* = 0.038; HIPP *P* = 0.044; and NAC *P* = 0.023). The administration of NOS inhibitors, L-NIL, and 7-NI, 30 min prior to administration of the soluble peptide, prevented this effect in all brain areas (Figure 2, Two-way ANOVA with Bonferroni correction, n.s.). No differences were found between Aβ-treated and SHAM rats in 5-HT content in all three areas investigated (data not shown), thus no pharmacological manipulation of nitrergic system was further carried out.

### Effects of Aβ administration and pharmacological manipulation on NOS expression

**Figure 3:**
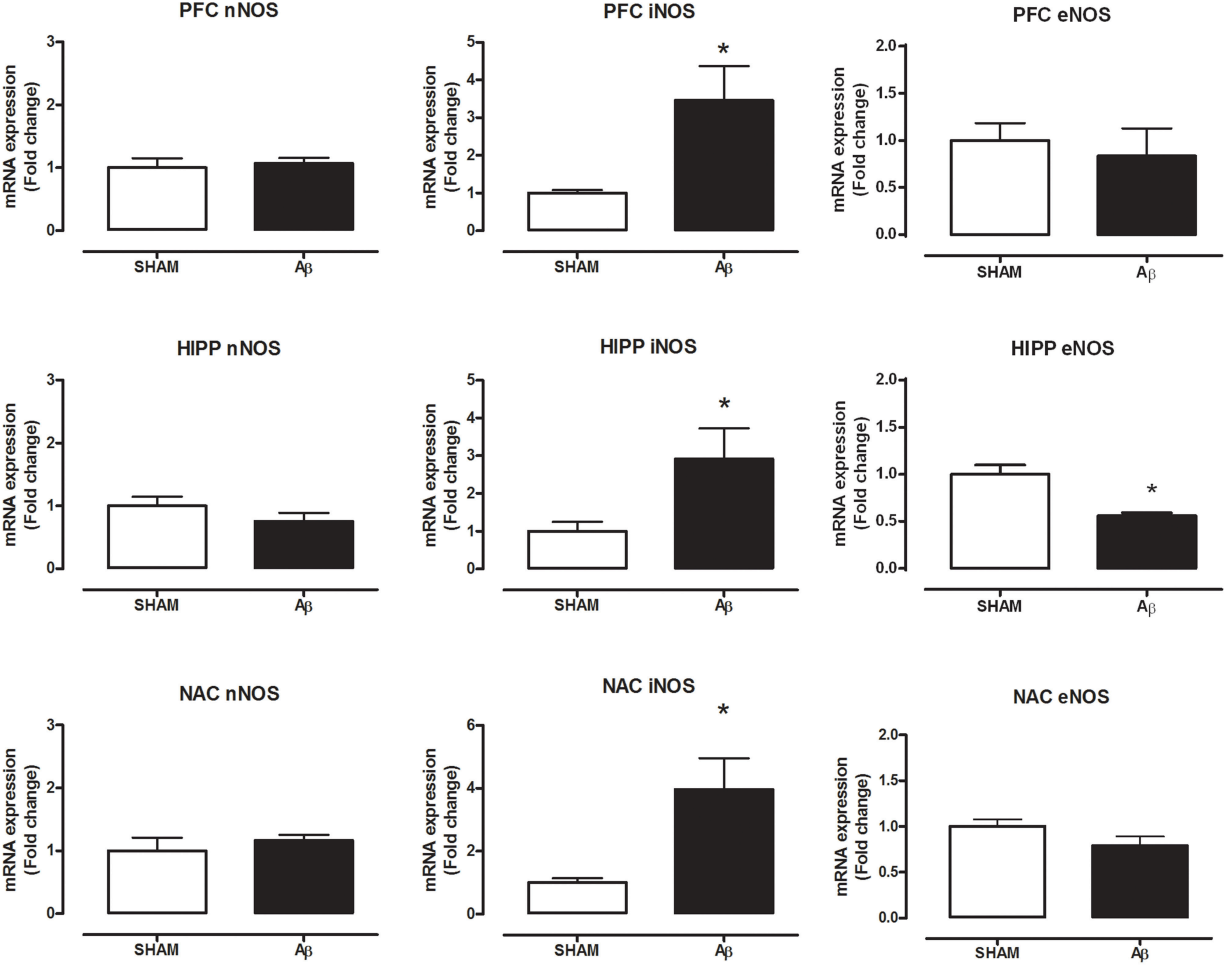
**Neuronal NOS, iNOS, and eNOS mRNA content in PFC, HIPP, and NAC of male Wistar rats 2 h after injection of water (5 μL i.c.v.; SHAM) or Aβ (4 μM, 5 μL i.c.v.; Aβ)**. Data are expressed as mean ± SEM (*n* = 6 per group; Student's *t*-test, ^*^P < 0.05).

**Figure 4:**
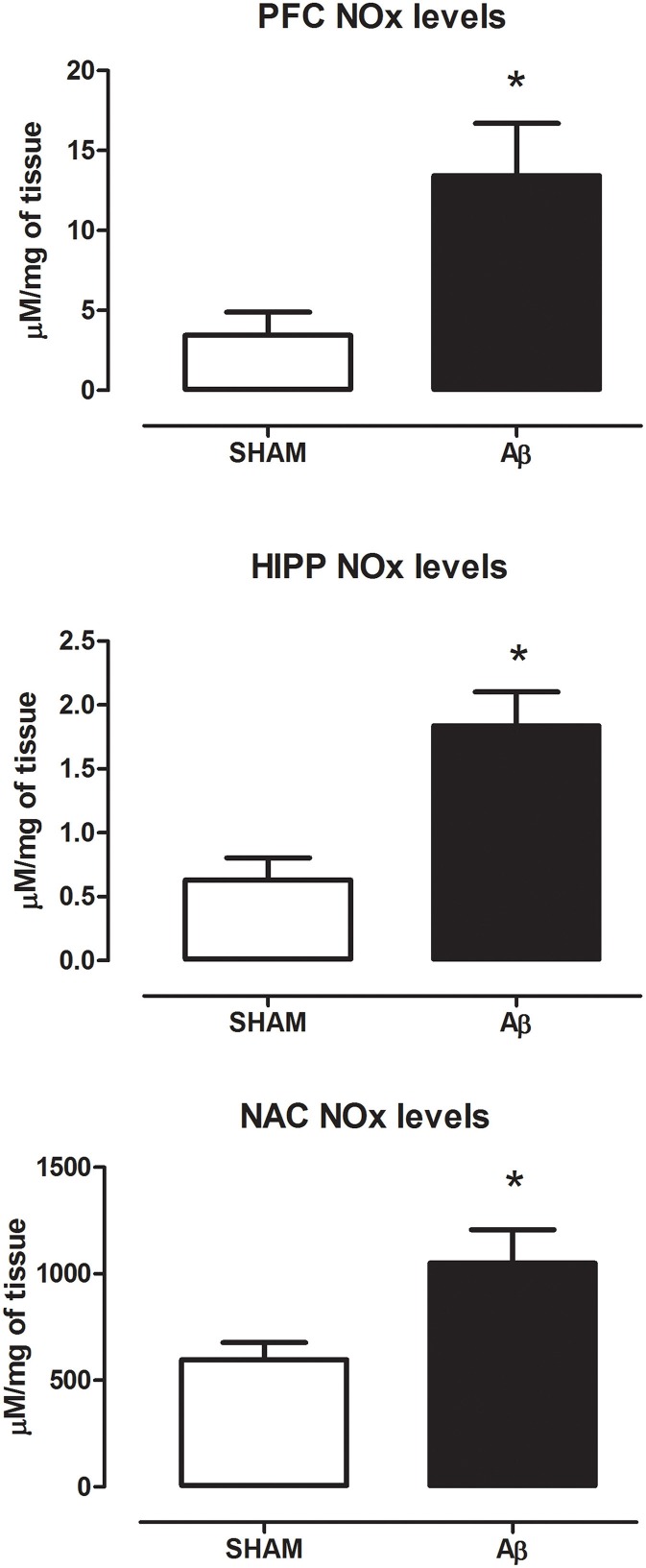
**NOx levels in PFC, HIPP, and NAC of male Wistar rats 2 h after injection of water (5 μL i.c.v.; SHAM) or Aβ (4 μM, 5 μL i.c.v.; Aβ)**. Data are expressed as mean ± SEM (*n* = 6 per group; Student's *t*-test, ^*^P < 0.05 for PFC and NAC and ^*^P < 0.01 for HIPP).

To investigate if Aβ administration was able to affect NOS expression, mRNA content of NOS enzymes (neuronal, endothelial, and inducible forms) was evaluated. A significant increase of iNOS was detected in the PFC (Student's *t*-test, *P* = 0.028), NAC (Student *t*-test, *P* = 0.041) and HIPP (Student's *t*-test, *P* = 0.031) of Aβ-treated rats, while nNOS was not affected in these brain areas (Figure 3). Moreover, results showed that eNOS mRNA was decreased only in HIPP (Student's *t*-test, *P* = 0.016), while no difference was evident in PFC and NAC (Figure 3). To evaluate iNOS activity, NOx levels were measured. Statistical analysis showed that the soluble peptide induced a strong increase in NOx levels in PFC (Student's *t*-test, *P* = 0.046), HIPP (Student's *t*-test, *P* = 0.003), and NAC (Student's *t*-test, *P* = 0.042; Figure 4).

### Effects of block of IL-1β receptors on Aβ-induced NE increase

**Figure 5:**
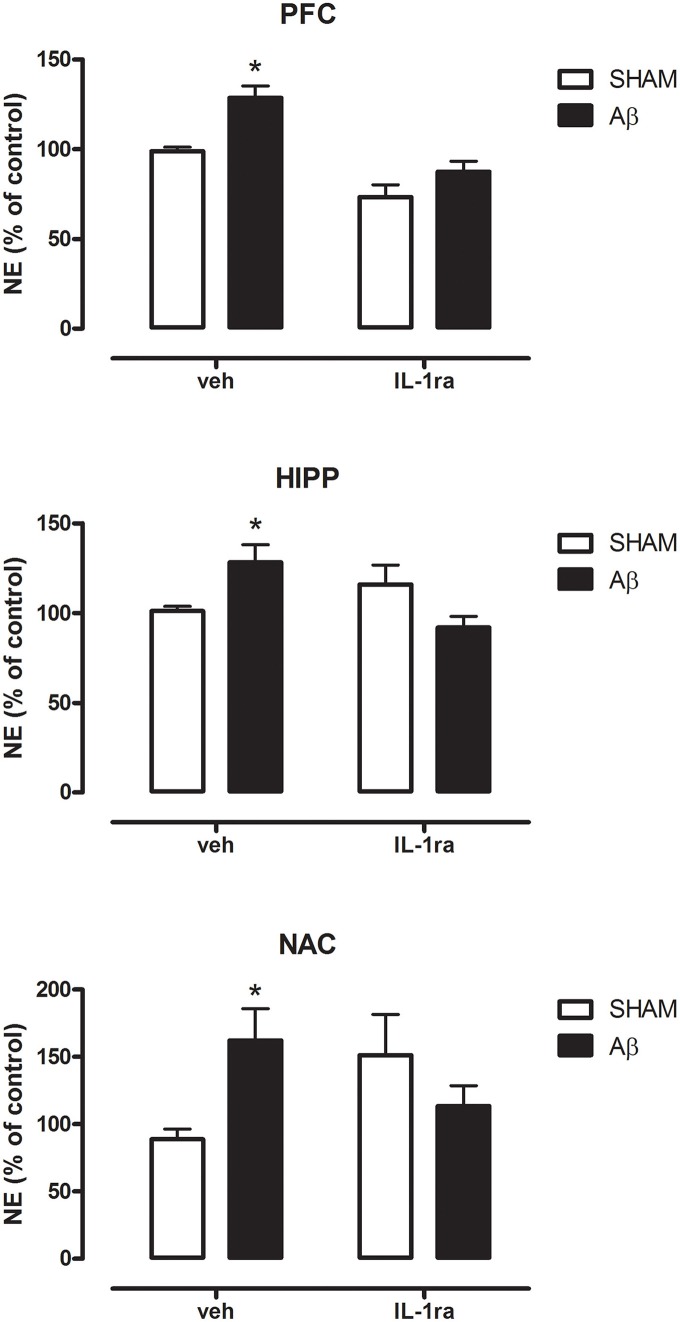
**NE levels in PFC, HIPP, and NAC of male Wistar rats 2 h after injection of water (5 μL i.c.v.; SHAM) or Aβ (4 μM, 5 μL i.c.v.; Aβ)**. Animals received 10 min before an i.c.v. injection of vehicle (saline, veh, 5 μl) or interleukin-1 receptor antagonist (IL-1ra, 100 ng 5 μl^−1^). Data are expressed as percentage of control (*n* = 5–6 per group; Two-way ANOVA with Bonferroni correction, ^*^P < 0.05).

In order to possibly establish a putative mechanism for the increase in NE Aβ-induced, we pre-treated the animals with the antagonist of IL-1 receptors, IL1ra. Results showed that in all investigated areas such treatment was able to prevent NE increase in Aβ-treated rats (Figure 5, Two-way ANOVA followed by Bonferroni post hoc test, n.s.).

## Discussion

In the present study, we found that a single i.c.v. injection of freshly prepared Aβ solution induced a significant increase of NE concentrations in the PFC, NAC, and HIPP when measured 2 h after administration. Moreover, treatment with nitrergic modulators and with the antagonist of IL-1 receptor prevented NE increase in Aβ-treated rats. We also found that the increase was accompanied with an increase in iNOS mRNA levels along with NOx concentrations in all brain areas investigated.

Although alterations in neurotransmitter secretion have been extensively reported in the case of other monoaminergic transmitters (Trabace et al., 2007; Preda et al., 2008; Morgese et al., 2014), the exact mechanism through which Aβ may alter NE concentrations in these areas is not completely understood yet. However, we hypothesized that the altered Aβ-induced NE content may be caused by messengers/neurotransmitters such as NO. The free-radical gas NO is produced by NOS and it modulates vital physiological functions (Moncada et al., 1991; Murad, 2003) acting as an intercellular messenger, which possess also neuromodulatory actions (Garthwaite, 1991; Snyder and Bredt, 1991). Patel and coworkers have demonstrated *in vitro* that, at hippocampal level, the activation of the noradrenergic system is neuroprotective in stressful condition and that, at least partially, these protective actions are mediated by NO signaling (Patel et al., 2010). Additionally, NE seems to control both enzymatic activity and expression of nNOS and iNOS in rat magnocellular neurons (Grange-Messent et al., 2004). In line with these observations, in the present study, we demonstrated that the increase in NE concentrations was accompanied with higher iNOS mRNA, along with increased NOx concentrations, suggesting that the effects of Aβ could be associated with NO-related actions on the noradrenergic system. Indeed, we have found that pharmacological inhibition of the nitrergic system, 30 min before Aβ injection, prevented the increase in NE concentrations. Our hypothesis that noradrenergic system activation could be mediated by NO release after NOS induction was supported by the observation that the increase in NE concentrations was prevented in Aβ-treated rats with the administration of different NOS inhibitors, 7-NI, and L-NIL. Indeed, the administration of both compounds completely prevented the increase in NE concentrations in all areas investigated, indicating that activation of NOS is necessary for this action to occur.

Furthermore, the altered release of NE may be explained through the release of other neuromodulators, such as cytokines. In this regard, Kamikawa and colleagues have reported that increased NE levels occur in mPFC via indirect glutamate release. In particular, the activation of IL-1β receptors at glutamatergic terminals leads to the release of glutamate acting at non-NMDA receptors in nerve terminals that induce NOS activation and then NO production (Kamikawa et al., 1998). We have previously demonstrated that an intra-hippocampal administration of Aβ evoked an increased release of IL-1β through a purinergic receptor (P2X7)-mediated mechanism (Sanz et al., 2009). Thus, we hypothesized that Aβ-dependent release of IL-1β may be able to generate such a cascade. Our hypothesis was pharmacologically confirmed, considering that blocking the IL-1 receptors inhibited the Aβ-induced increase of NE. Furthermore, a direct action of IL-1β on its receptors localized on glutamatergic terminals in the mPFC seems to be responsible for an initial release of NE after IL-1β injection (Kamikawa et al., 1998). This observation appears very interesting for our research, since we have recently demonstrated that, in a microdialysis study conducted in this same experimental model, Aβ was able to elicit an increase in the release of glutamate in PFC 2h after a single i.c.v. injection (Tucci et al., 2014).

On the other hand, many reports have indicated that Aβ directly interacts with the noradrenergic system and in particular, that Aβ directly binds to adrenergic receptors (Igbavboa et al., 2006; Wang et al., 2011). Wang et al. (2011) demonstrated, *in vitro* that Aβ may cause desensitization and subsequently internalization of β2 adrenergic receptors in prefrontal cortical neurons (Wang et al., 2011).

This increase in noradrenergic tone could also reflect a neuroprotective phenomenon. In this regard, it has been reported that NA can regulate glial activation and a valid approach for neurodegeneration could rely on pharmacological strategies leading to increase NA (Braun et al., 2014). Furthermore, *in vitro* studies have evidenced a protective effect of NA toward toxicity Aβ-induced by increasing neurotrophic factor expression via activation of β adrenergic receptor signaling cascade (Counts and Mufson, 2010; Liu et al., 2015). Reduced NA concentrations in LC projecting areas facilitates the inflammatory reaction of microglial cells after Aβ exposure, thus impairing microglial migration and phagocytosis, thereby decreasing Aβ clearance (Heneka et al., 2010). Interestingly, it has been reported that NA inhibits iNOS induction after inflammatory stimuli in astrocytes (Feinstein et al., 1993) and microglia (Dello Russo et al., 2004).

On the other hand, *in vitro* studies indicate that Aβ can alter neurotransmitter release by interacting with presynaptic proteins (Russell et al., 2012). In particular, the peptide was reported to be able to be internalized at presynaptic level where it can disrupt the complex synaptophysin/vesicle associate membrane protein 2 (VAMP2) leading to increased vesicle priming and exocytosis (Russell et al., 2012). NO can also interfere with neurotransmission considering that cGMP signaling and S-nitrosylation of proteins can cause enhanced presynaptic binding of syntaxin with VAMP and synaptosomal-associated protein-25 (SNAP 25) (Meffert et al., 1996). Therefore, we can hypothesize that the increased NA levels found in our model can be the result of a presynaptic interaction of Aβ and NO signaling molecules. Accordingly, it has been shown that NO recruitment may represent a compensatory mechanism to enhance synaptic transmission and plasticity in a transgenic model of early AD (Chakroborty et al., 2015).

Here we also found that Aβ was able to decrease eNOS mRNA only in HIPP. Such result is in agreement with literature data showing that Aβ reduces eNOS activity (Gentile et al., 2004). However, in our experience only HIPP seems to be affected. A possible explanation may rely on anatomical structure since HIPP is a close region to injection area. Moreover, it has been reported that eNOS deficient mice have higher Aβ levels in HIPP, indicating a putative interconnection in this area considering that NO supplementation reduced Aβ production (Austin et al., 2010, 2013). In this light, several reports indicated that the effect of Aβ may be region specific (Kar et al., 2004; Mura et al., 2012).

Ultimately, in our experimental conditions, we might possibly exclude a widespread toxic property of soluble Aβ on brain neurotransmitters. Indeed, 5-HT concentrations were not altered by Aβ administration in all investigated areas at this time-point, while we have previously found that in PFC the peptide reduced such neurotransmitter content 7 days after i.c.v. infusion, indicating that 5-HT impairment needs a longer time to occur (Colaianna et al., 2010). Furthermore, we have previously shown that i.c.v. injection or retrodialysis of Aβ leads to a significant reduction in DA concentrations in the PFC (Trabace et al., 2007). Thus, the effects of Aβ seem to be neurotransmitter-specific. Moreover, we have previously shown that the used concentrations of Aβ are not associated, at least acutely, with gross neurotoxicity as shown by Hoechst nuclear staining (Trabace et al., 2007). However, we cannot exclude, after Aβ treatment, the presence of more subtle signs of toxicity. In this regard, evaluation of sign of toxicity/apoptotic pathway activation could represent an interesting future field of research.

In conclusion, we have demonstrated that the noradrenergic system seems to be activated possibly as a compensatory mechanism following soluble Aβ increased levels. In particular, the modulation of nitrergic system and involvement of IL-1 receptors seem to play a crucial role in this process. Our results contribute to shed light on the physiological interplay among soluble Aβ, nitrergic and noradrenergic transmissions in the brain.

## Author contributions

MM and MC, wrote the paper, and performed experiments (animal surgery, post mortem NE quantification). EM, MZ, and SS performed experiments (PCR analyses and Nox quantification). PD, AH, VG, AD performed experiments (PCR analyses, AFM quantifications and TEM analyses). PC, VT, and PT analyzed the data. VC and LT revised the paper and designed the research study. All the authors critically revised the work and approved the final version.

### Conflict of interest statement

The authors declare that the research was conducted in the absence of any commercial or financial relationships that could be construed as a potential conflict of interest.

## Abbreviations

AD: Alzheimer's disease
Aβ: soluble beta amyloid
7-NI: 7-Nitroindazole
L-NIL: N6-(1-iminoethyl)-L-lysine-dihydrochloride
PFC: prefrontal cortex
HIPP: hippcampus
NAC: nucleus accumbens
NO: nitric oxide
iNOS: inducible Nitric oxide synthase
eNOS: endotelial Nitric oxide synthase
nNOS: neuronal Nitric oxide synthase
NOx: nitrates and nitrites
NE: norepinephrine
IL-1: interleukin 1
IL-1ra: interleukin 1 receptor antagonist.
